# Damage Detection in a Polymer Matrix Composite from 4D Displacement Field Measurements

**DOI:** 10.3390/ma16186300

**Published:** 2023-09-20

**Authors:** Ana Mandić, Viktor Kosin, Clément Jailin, Zvonimir Tomičević, Benjamin Smaniotto, François Hild

**Affiliations:** 1 Faculty of Mechanical Engineering and Naval Architecture, University of Zagreb, 10000 Zagreb, Croatia; 2 Université Paris-Saclay, CentraleSupélec, ENS Paris-Saclay, CNRS, LMPS–Laboratoire de Mécanique Paris-Saclay, 91190 Gif-sur-Yvette, France; 3 Institut für Angewandte Mathematik (IfAM), Leibniz Universität Hannover, 30167 Hannover, Germany; 4 GE HealthCare, 78530 Buc, France; clement.jailin@ge.com

**Keywords:** polymer matrix composite, projection based digital volume correlation, damage growth, correlation residuals

## Abstract

Standard Digital Volume Correlation (DVC) approaches enable quantitative analyses of specimen deformation to be performed by measuring displacement fields between discrete states. Such frameworks are thus limited by the number of scans (due to acquisition duration). Considering only one projection per loading step, Projection-based Digital Volume Correlation (P-DVC) allows 4D (i.e., space and time) full-field measurements to be carried out over entire loading histories. The sought displacement field is decomposed over a basis of separated variables, namely, temporal and spatial modes. In the present work, the spatial modes are constructed via scan-wise DVC, and only the temporal amplitudes are sought via P-DVC. The proposed method is applied to a glass fiber mat reinforced polymer specimen containing a machined notch, subjected to in situ cyclic tension and imaged via X-ray Computed Tomography. The P-DVC enhanced DVC method employed herein enables for the quantification of damage growth over the entire loading history up to failure.

## 1. Introduction

As it provides 3D images of scanned microstructures in a non-destructive way, X-ray Computed Tomography (XCT) has given a major impetus to the field of mechanics of materials [1,2]. With the recent developments of testing machines, 3D full-field displacement fields can be quantified in situ by coupling XCT and Digital Volume Correlation (DVC) into unique frameworks [3,4]. As DVC is based upon matching the gray levels between fully reconstructed volumes, the major limitations of such approach are its low temporal sampling and experiment duration. Each reconstructed volume requires from few minutes up to few hours of radiograph series to be acquired. This process governs the number of possible acquired scans. As the material response between two consecutive scans is not accessible, the quantification of time-dependent phenomena (e.g., stress-relaxation or crack propagation) is restricted. Furthermore, due to relaxation during in situ experiments, the reconstructed volumes may be impacted by motion artifacts. This issue can be avoided by starting the radiograph acquisition only after the measured force has stabilized. The aforementioned restrictions may be overcome by two different routes. The first route is to perform fast acquisitions, say in synchrotron facilities, with extremely bright X-ray beams using high-speed cameras. However, synchrotron imaging presents some drawbacks such as poor accessibility, high doses and the reconstruction quality that may degrade due to blur induced by vibrations [5,6]. Another route is to utilize the recently developed Projection-based Digital Volume Correlation (P-DVC), which aims to surpass the low temporal resolution attributed to classical (i.e., scan-wise) DVC approaches [7]. This method enables for the measurement of 4D (i.e., space and time) displacement fields from series of 2D radiographs acquired at different angles and load levels (instead of working with series of reconstructed 3D volumes). In such a way, huge gains in acquisition time and testing duration can be reached [4,8,9].

The ever-increasing application of fiber-reinforced polymers (FRPs) across various industrial branches is attributed to their advantageous properties such as high stiffness-to-weight ratios, which outperform conventional engineering materials [10]. Due to the occurrence of various damage mechanisms at different scales under mechanical loading, XCT is appealing in damage analyses of FRPs. The latter has been employed to quantify damage growth in FRPs under various loading conditions by, e.g., analyzing volumes [11,12,13,14]. However, it is worth noting that with this approach, only microstructural changes were evaluated, while it cannot provide access to the bulk kinematics, nor can it correlate it with microstructural changes. This limitation can be surpassed by DVC, which enables for 3D full-field displacement measurements [15,16]. Local approaches to DVC, which do not assume continuity of the displacement fields as they split the investigated Region of Interest (ROI) into smaller zones and register them independently, have been employed in the analysis of FRPs [17,18,19]. Conversely, global approaches based on, e.g., finite element (FE) discretizations were introduced more recently [20,21]. Such approaches are based on the assumption of continuity of displacement fields. A significant advantage of FE-DVC is the availability of correlation residuals. They correspond to the gray level differences between the reference volume and that of the deformed material corrected by the measured displacement field [4]. The conservation of gray levels is the underlying hypothesis of DVC [16,22,23]. Therefore, the correlation residuals can be used to check the quality of the registration. The initiation and growth of damage violates gray level conservation, thus the analysis of correlation residual fields (for converged displacement fields) reveal discontinuities corresponding to damaged zones [4,24,25]. Furthermore, FRPs are likely to undergo stress relaxation during tests [26,27]. As reconstructed volumes may last for up to hours, the specimen should be in a steady state to avoid poor image quality due to motion artifacts. Therefore, performing full-field displacement measurements in space and time is crucial especially for FRPs. The full insight into the material behavior is then provided in situ, and it is fully coupled with changes in the underlying microstructure.

In general, P-DVC relies on spacetime discretizations of the sought displacement fields [9]. A new spacetime framework was recently proposed to measure 4D displacements by combining DVC and P-DVC analyses [28]. By using spatiotemporal separations of variables, the spatial components were obtained via scan-wise DVC, and the *instantaneous* temporal amplitude was computed via P-DVC on each individual radiograph acquired on-the-fly during the whole loading history. In the following study, a new P-DVC enhanced DVC algorithm is introduced in which spatial modes are measured via DVC, and the temporal modes are sought with P-DVC. For the first time, the temporal modes are constructed such that they are compatible with the loading history of the experiment. In this regard, temporal shape functions are introduced. The displacement fields are sought in a 4D (i.e., 3D in space and 1D in time) vector space generated by a reduced spacetime kinematic basis. Only one projection per loading level is still needed. Additionally, different temporal interpolations are investigated, and the number of spatial modes selected to describe the specimen kinematics is analyzed and justified.

The aim of this work is the 4D characterization of a mat glass fiber reinforced polyester resin subjected to in situ cyclic tension, and imaged via XCT. A dogbone specimen contained a machined rectangular notch to induce high strain gradients. The properties and fabrication procedure of the investigated composite material are presented in Section 2, followed by a description of experimental setup. In Section 3, the principle of the proposed procedure is presented by introducing DVC and P-DVC formulations. The method is then applied to an in situ cyclic tensile test of a single-notched specimen (Section 4). The reconstruction error was first quantified by evaluating the difference between the corresponding projections and re-projections of the reconstructed reference volume. Last, the methodology to quantify damage growth from projection residual fields is presented. The implemented methodology is employed to directly assess damage from radiographs, thereby representing a completely novel approach.

## 2. Material and Methods

### 2.1. Material and Experimental Setup

**Figure 1:**
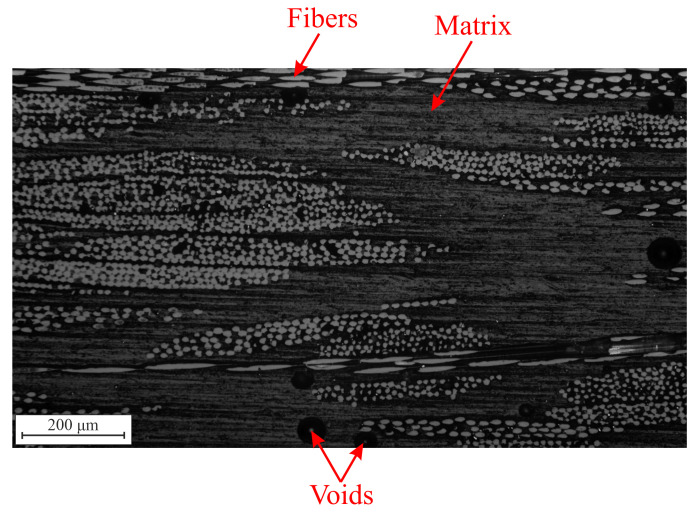
Optical micrograph of the glass fiber mat reinforced polyester resin composite studied herein.

In the present work, a polyester resin reinforced with a continuous glass fiber mat is investigated. The composite plate comprised 12 layers of R-glass fiber mat and was produced by manual lay-up, followed by compression molding. In terms of volume fraction, the composition had 40% of fibers, 55% of resin and 5% of voids (due to the fact that vacuum was not applied during molding). Figure 1 shows an optical micrograph of the investigated material. Due to the contrast between constituents, this material is well-suited for DVC, which relies on natural contrast [4].

**Figure 2:**
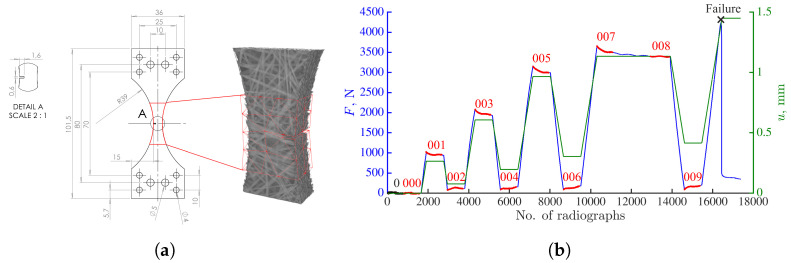
(**a**) Geometry of the investigated single notched dogbone specimen together with the Region of Interest (depicted with red contour) and finite element mesh employed herein (superimposed over the reconstructed reference volume). The dimensions are expressed in mm. The size of Region of Interest was 384×384×486 vx, while the physical length of one voxel was 58 µm. (**b**) Measured uniaxial force (blue) and stroke history (green) of the studied in situ tensile test. The black dots mark the acquisition of the high-quality reference scan (0). The red dots depict the load levels at which full volumes were reconstructed. These volumes were employed in the scan-wise DVC analyses.

The thickness of the investigated specimen was 5.2 mm. The specimen contained a machined rectangular notch (Figure 2a), which induced high strain gradients. The depth of the notch was 1.6 mm, while its width was 0.6 mm. The in situ cyclic tensile test was performed with the TTC Deben testing machine, i.e., the specimen was continuously loaded, rotated and imaged in the X50+ scanner (North Star Imaging) of LMPS. Before prescribing any loading, a reference scan (denoted with the black dots in Figure 2b), comprising 800 projections was acquired at equally spaced angles spanning over a complete 360° revolution with high-quality (HQ) scanning parameters (Table A1 in Appendix A). As this scan took approximately 2 h to be completed, the number of averaging frames was subsequently reduced from 20 to 1 to lower the experiment duration and mitigate, e.g., stress-relaxation. Such scanning parameters are called continuous scanning, and each scan took 4 min to be completed. After the acquisition of the reference scan, cyclic tension was applied at a constant stroke velocity of 4 µm/s. The radiographs were continuously acquired during the entire loading history at 4 frames per second with angular increments of 0.47º. These settings led to 768 radiographs per turn. It is worth highlighting that the P-DVC methodology presented herein is not dependent on the rate of the conducted experiments. However, there may be limitations related to the temporal resolution of each imaging device.

The deformed scans were reconstructed from the stages where a constant stroke was applied (marked with red dots in Figure 2b). They were used to measure 3D displacement fields via FE-DVC within the Correli 3.0 framework [29]. The FE mesh used in all DVC analyses (Figure 2a) was composed of first-order tetrahedral (T4) elements with piecewise linear (P1) shape functions. It was made of 88 nodes (with three DOFs per node), and the mean element length was equal to 12 vx.

A total of 17,442 projections was acquired during the prescribed loading history. The radiographs had an initial definition of 1944 × 1536 px. The focus was put on the notched-region (as the majority of damaged zones concentrated within this region [27]). Coarse graining of 4×4 elementary pixels into one superpixel was performed. This led to 486×384 px radiographs (called images at scale 4). The physical length of one voxel was equal to 58 µm. The volumes were reconstructed within the ASTRA toolbox with the simultaneous iterative reconstruction (SIRT) algorithm suited for cone beams [30], and employing geometrical parameters given by the tomograph calibration.

### 2.2. Projection-Based Digital Volume Correlation

This section outlines the basic principles of DVC and P-DVC. The notations used herein are introduced as well. The radiographs (i.e., the sum of absorption coefficient along each material point **x** of the beam ray hitting the detector at position **r**) are denoted as p(r,t) at different time *t*. The reconstructed volume is such that its projection should match the recorded projection p(r,t), which results in a linear relationship between *f* and *p*

(1) $$\Pi_{\theta (t)}\left[f\left(x\right)\right]=p\left(r,t\right),$$

where $\Pi_{\theta (t)}$ is the projection operator at angle θ(t). The reconstruction is thus the inversion of this linear system for a large sequence of angles [31]. By reconstructing a series of such tomographic images, the 3D kinematics of a medium can be quantified. The displacement field is estimated via DVC, which relies on the conservation of gray levels

(2) $$f^{T_{0}}\left(\hat{x}\right)=f^{T}\left(\hat{x}+\hat{U}\left(\hat{x},T\right)\right),$$

where $\hat{x}$ denotes the position of any material point in the reference configuration, and $\hat{U}$ the measured displacement field that describes the change of the reference volume $f^{T_{0}}$ (usually captured in the undeformed state) to the deformed configuration (here denoted as $f^{T}$). Two different time scales have to be highlighted here. First, the 3D images are reconstructed over the duration needed for one turn *T*. Hundreds up to thousands of instantaneous radiographs acquired at θ(t) are needed over a full revolution (or turn *T*) of the specimen. The turn parameter *T* thus denotes the different stages at which a 3D scan was captured, while $T^{0}$ denotes that of the reference scan. The displacement field to be measured is obtained from the minimization of $\Gamma_{\mathrm{DVC}}$, which is the quadratic differences between the corrected deformed and the reference volumes

(3) $$\Gamma_{\mathrm{DVC}}=\sum_{\hat{x}}{\left(f^{T}\left(\hat{x}+{\hat{U}}^{\mathrm{DVC}}\left(\hat{x},T\right)\right)-f^{T_{0}}\left(\hat{x}\right)\right)}^{2}$$

with respect to the parameterization of the trial displacement fields ${\hat{U}}^{\mathrm{DVC}}$. A global (i.e., FE-based) approach was utilized in this work. The displacement field is then expressed in a kinematic basis consisting of FE shape functions ${\hat{\Psi }}_{j}$

(4) $${\hat{U}}^{\mathrm{DVC}}\left(\hat{x},T\right)=\sum_{j}v_{j}\left(T\right){\hat{\Psi }}_{j}\left(\hat{x}\right),$$

where $v_{j}$ are the nodal displacements. As the sought displacement fields are determined with respect to the reference configuration, the DVC framework presented herein is Lagrangian.

Instead of working with fully reconstructed volumes, P-DVC measures 3D displacement fields from a series of 2D radiographs captured at different angles θ(t) and loading steps F(t) [32]. The cost function $\Gamma_{P\text{-}\mathrm{DVC}}$ to be minimized is defined as follows

(5) $$\Gamma_{P\text{-}\mathrm{DVC}}=\sum_{r,t}{\left(\Pi_{\theta (t)}\left[f^{T_{0}}\left(x-u\left(x,t\right)\right)\right]-p\left(r,t\right)\right)}^{2}.$$

According to Equation (5), the problem is no longer stated in the Lagrangian framework, but in the Eulerian system (contrary to Equation (4)). To complete the notations needed to describe the motion in Lagrangian and Eulerian settings, it is worth highlighting that the displacement at any spatial location *x* reads

(6) $$u\left(x,t\right)=x-\hat{x}=\hat{u}\left(\hat{x},t\right).$$

The present P-DVC framework requires the acquisition of one reference volume $f^{T_{0}}$ from which the microstructure of the sample is known. The remaining unknowns are the displacements sought in the spatiotemporal framework. In the present study, the spatial components are constructed with all scan-wise DVC results, while only temporal modes are sought via P-DVC

(7) $$u\left(x,t\right)=\sum_{\tau }\alpha_{\tau }{\hat{U}}^{\mathrm{DVC}}\left(\hat{x},\tau \right)\sigma_{\tau }\left(t\right),$$

where $\sigma_{\tau }\left(t\right)$ denote the temporal modes, and $\alpha_{\tau }$ the temporal amplitudes. The presented methodology is 4D in the sense that it gives access to the entire displacement field u(x,t) in (3D) space and (1D) time. Furthermore, DVC fields are the result of the minimization (Equation (3)) when the displacement fields are parameterized with FE shape functions (Equation (4)).

The displacement fields are sought in a vector space generated by a reduced kinematic basis. The interpolation functions $\sigma_{\tau }$ naturally introduce temporal regularization [9]. The nodal displacements $v_{j}\left(\tau \right)$ are measured via FE-DVC, and the temporal amplitudes gathered in column vector {α} are sought via P-DVC and iteratively updated

(8) $$\left\{\alpha^{l+1}\right\}=\left\{\alpha^{l}\right\}-\left\{\delta \alpha^{l}\right\},$$

where the corrections are calculated by performing Gauss-Newton minimizations of the functional $\Gamma_{P\text{-}\mathrm{DVC}}$

(9) $$\left\{\delta \alpha^{l}\right\}={\left[M^{l}\right]}^{-1}\left\{m^{l}\right\}.$$

In Equation (9), the Hessian matrix $M_{\tau k}$ is updated for each iteration *l*

(10) $$M_{\tau k}^{l}=\sum_{r,t}\left(S_{\tau }^{l}\left(r,t\right)\sigma_{\tau }\left(t\right)\right)\;\cdot \;\left(S_{k}^{l}\left(r,t\right)\sigma_{k}\left(t\right)\right),$$

where $S_{\tau }^{l}$ is the projected sensitivity at iteration *l*

(11) $$S_{\tau }^{l}\left(r,t\right)=\Pi_{\theta (t)}\left[{\hat{U}}^{\mathrm{DVC}}\left(\hat{x},\tau \right)\;\cdot \;\nabla f^{T_{0}}\left(x-u^{l}\left(x,t\right)\right)\right].$$

Furthermore, the second member vector $m_{\tau }^{l}$ is based on the projection residual fields

(12) $$m_{\tau }^{l}=\sum_{r,t}S_{\tau }^{l}\left(r,t\right)\sigma_{\tau }\left(t\right)\varphi_{C}^{l}\left(r,t\right),$$

where the projection residual field per angle at each iteration *l* reads

(13) $$\varphi_{C}^{l}\left(r,t\right)=p\left(r,t\right)-\Pi_{\theta (t)}\left[f^{T_{0}}\left(x-u^{l}\left(x,t\right)\right)\right].$$

According to Equation (13), the reference volume, corrected by the displacement field $u^{l}\left(x,t\right)$, and projected for each angle θ(t) should match the acquired projection p(r,t). The projection residual field for all angles is to be minimized during the iterative procedure. As in DVC, these residuals reveal what was not captured by the 4D corrections, herein projected onto the 2D detector plane. A general overview of 4D analyses in which spatial modes are controlled by DVC fields is shown in Algorithm 1.
**Algorithm 1:** P-DVC with DVC spatial modes Select spatial modes ${\hat{U}}^{\mathrm{DVC}}\left(\hat{x},\tau \right)$ Choose an initial guess of $\left\{\alpha^{0}\right\}$ **while***$∥\left\{\delta \alpha^{l}\right\}∥>10^{-3}$***do**(  Corrections $f^{l}\left(x,t\right)\leftarrow \;f^{T_{0}}\left(x-u^{l}\left(x,t\right)\right)$  Update $\left[M^{l}\right]$ and $\left\{m^{l}\right\}$  Solve $\left\{\delta \alpha^{l}\right\}={\left[M^{l}\right]}^{-1}\left\{m^{l}\right\}$  Update temporal amplitudes $\left\{\alpha^{l+1}\right\}=\left\{\alpha^{l}\right\}-\left\{\delta \alpha^{l}\right\}$  Update displacement fields $u^{l+1}\left(x,t\right)=\sum_{\tau }\alpha_{\tau }^{l+1}{\hat{U}}^{\mathrm{DVC}}\left(\hat{x},\tau \right)\sigma_{\tau }\left(t\right)$  Update projection residuals $\varphi_{C}^{l+1}\left(r,t\right)=p\left(r,t\right)-\Pi_{\theta (t)}\left[f^{T_{0}}\left(x-u^{l+1}\left(x,t\right)\right)\right]$ **end**(

## 3. Results

In this section, the previous P-DVC enhanced DVC framework is applied to an in situ cyclic tensile test on a single-notched specimen. First, the reconstruction error of tomographic volumes is evaluated. The results of 4D measurements are then presented. All presented analyses converged, i.e., the norm of the change of amplitude corrections {δα} (Equation (9)) between two iterations became less than $10^{-3}$. Convergence and the obtained temporal amplitudes are discussed.

### 3.1. Evaluation of Reconstruction Error

According to Equation (13), the P-DVC algorithm minimizes the projection residual fields (i.e., differences between the acquired radiograph p(r,t) and the reference volume deformed by the measured displacement field, and projected according to the angle θ(t)). This procedure is herein extended to the initial step (i.e., the reference HQ scan 0), where u=0. The reconstruction error was quantified by evaluating the difference between the corresponding projections and re-projections of the reconstructed reference volume 0. Due to acquisition noise, uncertainties in the geometric parameters used for the reconstruction and assumptions in the projection operator (e.g., pixel/voxel integration, beam hardening), the initial projection residual field was not equal to 0.

**Figure 3:**
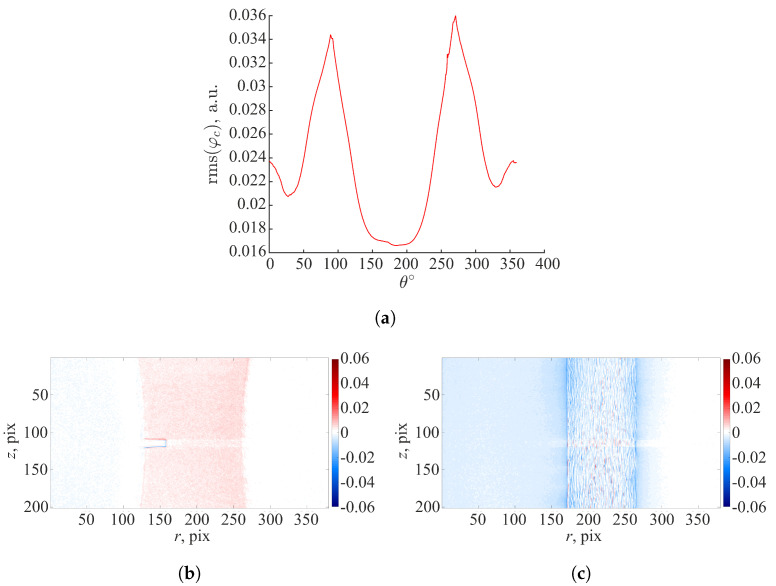
(**a**) Root mean square (rms) projection residuals evaluated for the first 360° revolution (scan 0). Initial projection residual field for $\theta =0^{\circ }$ (**b**) and $\theta =90^{\circ }$ (**c**).

The root mean square (rms) of projection residuals ($\varphi_{C}$), expressed in arbitrary units, evaluated for the first 360° revolution (scan 0) is shown in Figure 3a. The projection residuals within the whole ROI are low with respect to the mean dynamic range of the original radiographs (i.e., 0.7 a.u.). The highest residual levels are reached for $\theta =90^{\circ }$ and $\theta =270^{\circ }$. Figure 3b,c shows the projection residuals for $\theta =90^{\circ }$ and $\theta =270^{\circ }$. These initial projection residual maps are shown with a divergent color map to highlight positive and negative values. The highest rms($\varphi_{C}$) levels (i.e., $\theta =90^{\circ }$ and $\theta =270^{\circ }$) are due to the specimen orientation with respect to the X-ray source. The thickness of the specimen with respect to the X-ray source is the highest at those angles, which may introduce nonlinearity in the X-ray attenuation. The initial projection residual field for $\theta =0^{\circ }$ reveals elevated levels close to the edges of the notch. The present volumes were reconstructed with the SIRT algorithm, which iterates between forward and backprojections until it reaches convergence. The algorithm was initialized with the Feldkamp–Davis–Kress (FDK) procedure. SIRT is computationally more expensive compared to FDK. However, it provides images with reduced noise. In the present case, the volumes were reconstructed with 100 SIRT iterations. The reconstruction residuals may be reduced by further adjusting the geometric parameters of the tomographic setup, and increasing the number of SIRT iterations. Other sources for such errors could be small motions during scanning and beam hardening, which were not corrected in the present work.

### 3.2. Full-Field Measurements over the Entire Loading History

**Figure 4:**
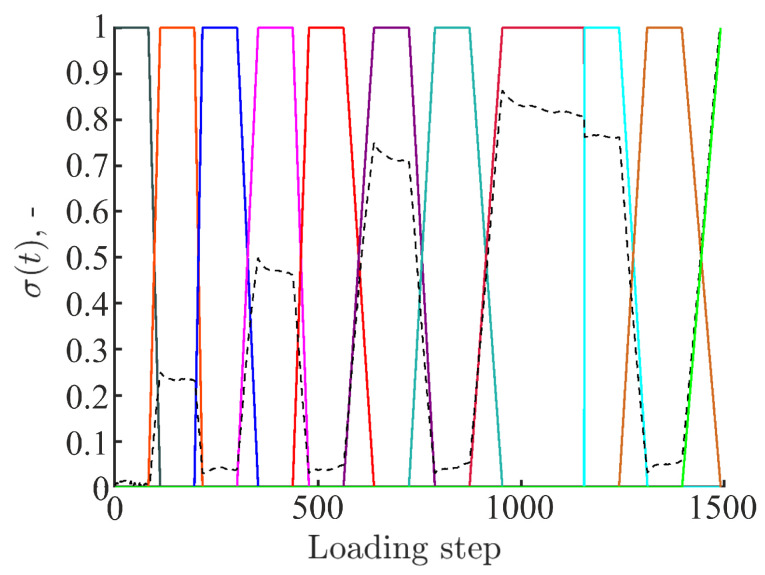
Temporal modes consisting of 11 plateau-like functions. The black dashed line represents the measured force signal normalized by its maximum value.

All the results presented in the sequel are obtained with a temporal basis consisting of 11 plateau-like functions interpolating the cyclic loading history (Figure 4). The selection of this temporal basis is detailed in Appendix B. The temporal shape functions are linked to 11 spatial modes. The latter ones are constructed via DVC, i.e., by performing volumetric correlations between scans 0 (reference scan) and all subsequent reconstructed volumes (Figure 2b). It is important to emphasize that all P-DVC measurements are performed until specimen failure (i.e., *after* acquiring scan 009). The last temporal mode is related to the DVC field of scan 007. The (small) force drop between scans 007 and 008 was due to the fact that the continuous acquisition between these two steps was interrupted by acquiring one high quality scan, which was excluded from the present analyses.

The projections to be registered with P-DVC are selected so that the angle between two consecutive acquisitions is approximately equal to 4.6°. This choice results in 1491 projections to be analyzed (out of a total of 17,442 acquired during the complete loading history). The chosen temporal sampling leads to 77 projections per turn (360° revolution). The sampling is governed by the computation time. In P-DVC procedures, the heaviest operations are the computation of the Hessian matrices and the deformation of the reference volume at each time step. To perform measurements in reasonable time, such trade-off had to be made between temporal resolution and computation time. Compared to standard DVC analyses that would yield 11 fields in the present analyses, the full P-DVC analysis probes 1491 radiographs corresponding to different temporal states (i.e., more than 2 orders of magnitude higher than regular DVC).

**Figure 5:**
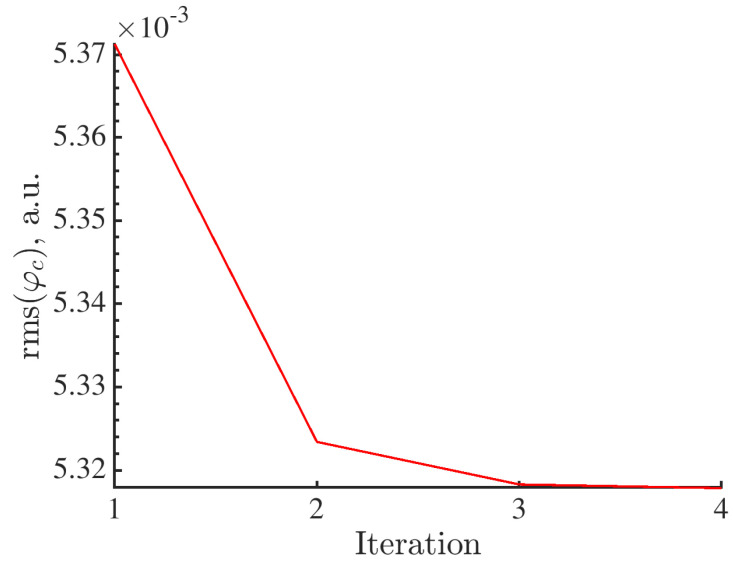
Change of rms projection residuals over the entire Region of Interest during the iterative procedure.

The initial values in the amplitude vector $\left\{\alpha^{0}\right\}$ are set to 1 (Equation (7)). The P-DVC analysis takes 4 iterations to converge. Each iteration lasts approximately 5 h. The measured displacements yield a global decrease of the residuals within the investigated ROI as shown in Figure 5.

The converged temporal amplitudes are gathered in Table 1. They are all close to 1, except for the last one where the amplitude is greater than 1. This is due to the fact that the DVC displacement field of scan 007 (Figure 2b) is used as a spatial component of the last mode, while 4D kinematic measurements are performed up to specimen failure.

Furthermore, the rms differences between the 3D nodal displacements obtained with FE-based DVC and projection-based measurements are calculated. Since large RBRs in *x* and *y* directions are observed during the experiment, these differences are only calculated for *z*-displacements as the mechanical component is dominant in that direction. The rms differences are computed between the DVC displacement fields and converged P-DVC displacement fields for the time-steps corresponding to the beginning of each constant stroke stage. The corresponding values are gathered in Table 2. As the projections acquired at the maximum load levels are affected by time-dependent motions (i.e., stress-relaxation phenomena), which influence the reconstruction of the full volume, differences are expected. From the values reported in Table 2, it is concluded that, when employing 11 plateau-like temporal functions, the rms differences are very small. With such settings, the kinematics of the specimen was properly captured.

## 4. Discussion: Quantification of Damage Growth

**Figure 6:**
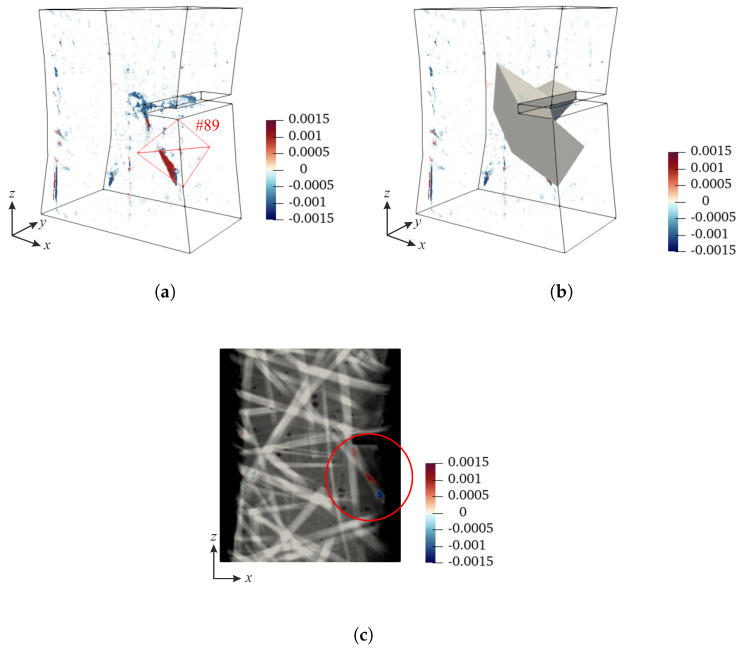
(**a**) DVC residuals of scan 007. The red contour denotes element #89. (**b**) Finite elements containing the highest DVC residual levels. (**c**) DVC gray level residuals laid over the mesostructure section of the front specimen surface. The red circle marks the area exhibiting elevated correlation residuals within element #89.

In this section, the methodology to quantify damage growth from projection residual fields is outlined and discussed for one particular case. The validation of the proposed procedure is detailed in Appendix C. The starting point of the method is to exploit the fact that DVC residuals revealed damaged zones within the specimen. Figure 6a shows the DVC residual field of scan 007 (last loading plateau prior to specimen failure). This map reveals numerous damaged zones in the specimen bulk, the majority of which are concentrated within the notched region. Next, the absolute maximum value of correlation residuals within the whole ROI is determined. The following criterion is employed to find finite elements that have correlation residuals greater than 70% of the maximum value integrated over each element volume

(14) $$\mathrm{rms}{\left(\varphi_{C,\mathrm{DVC}}\right)}^{\mathrm{Mask}}>0.7\;\cdot \;\mathrm{rms}{\left(\varphi_{C,\mathrm{DVC}}\right)}^{\mathrm{ROI},\max}$$

Based on the previous criterion, 14 elements are found. These elements compose the mask shown in Figure 6b, which is used to inspect the projection residuals. The mask is projected onto the detector plane with the projection operator $\Pi_{\theta (t)}$. The quantification of damage growth is performed within each element of the mask.

The analysis of damage growth is performed within element #89, which is located below the notch root (marked with red contour in Figure 6a. This element contains the zone of elevated correlation residuals, which correspond to a crack propagating from the notch root (Figure 6c).

**Figure 7:**
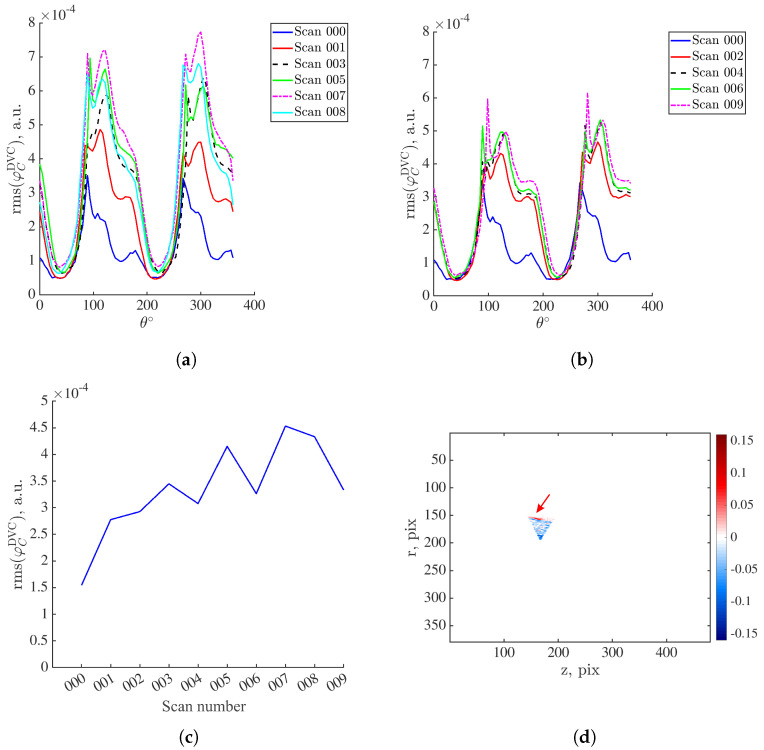
(**a**) Rms DVC residuals of element #89 projected onto the detector plane computed for the scans acquired at the peak of the loading cycles (Figure 2b). (**b**) Corresponding residuals computed for the scans acquired at the unloaded steps (Figure 2b). (**c**) Rms DVC residuals of element #89 during the entire turns for scan acquisitions. (**d**) Projected DVC residuals of element #89 when $\theta =75^{\circ }$. The red arrow marks the crack visible for this specific angle.

As 1491 radiographs are analyzed with P-DVC, not all angles are inspected in the damage procedure. To find for which angles damage (i.e., mesocrack) is most pronounced, the DVC residuals within element #89 are projected onto the detector plane. First, the rms residuals are computed for the full specimen revolution for both loaded and unloaded plateaus. The residuals of the loaded scans are shown in Figure 7a, whereas those of the unloaded acquisitions in Figure 7b. The rms residual levels of scan 000 (acquired in the undeformed state) is included in order to track the increases in DVC residuals with respect to the base level. For scan 001, there is an increase in correlation residuals with respect to levels of scan 000. When the specimen is unloaded (scan 002), the residuals remain close to the levels of scan 001. This is trend is also visible when plotting the rms DVC residuals of element #89 during the entire returns for the acquisitions of scans 001 and 002. These increases in correlation residuals compared to the base level are attributed to the specimen kinematics [27] and damage still did not occur at this stage.

As the specimen is once again loaded (scan 003), the correlation residuals keep increasing. Upon subsequent unloading, the residual levels decrease and are very close to those of scan 002. Damage growth did not yet occur, or if it had, it remained very low and at a scale lower than the scan resolution [33]. The significant increase in the correlation residuals compared to previous acquisitions is visible for scan 005. This is especially pronounced in Figure 7c. At this stage, the crack originating from the notch root and propagating below it is visible on DVC correlation residual field [34]. The subsequent unloading (scan 006) led to partial crack closure (as the correlation residuals were higher than those of the previous unloaded acquisitions, Figure 7c). At the peak of the last loading plateau prior to specimen failure (scans 007 and 008), the correlation residuals once again increase, and upon specimen unloading (scan 009), partial crack closure appears. From the reported data, the first interval of residual increase is within the range of 50° to 110°. In this range, damage is expected to be pronounced on residual fields. Thus, the projected DVC residuals within element #89 are shown in Figure 7d for an angle of $75^{\circ }$. The area containing elevated residuals corresponding to the crack is marked with the red arrow.

**Figure 8:**
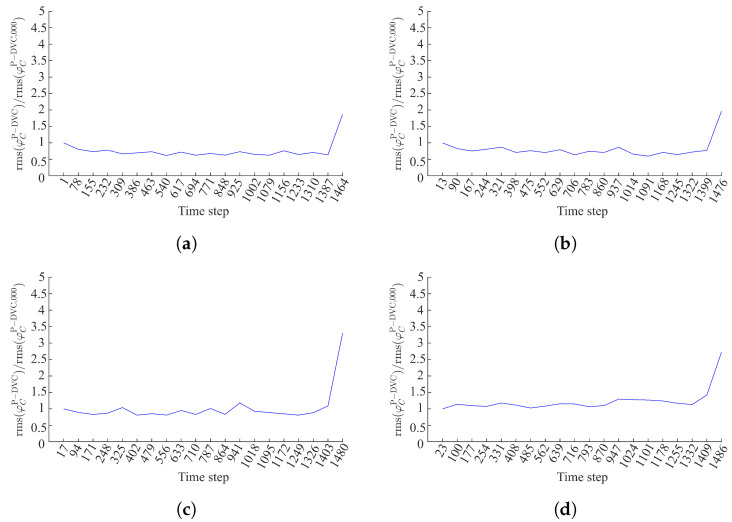
Amplification of projection residuals within element #89 when (**a**) θ=0°, (**b**) θ=56°, (**c**) θ=75° and (**d**) θ=105°.

With the previous observations, the projection residuals are only inspected for specific angles, namely, 0 (to prove that this angle is damage-insensitive for this specific case), 56°, 75° and 105° acquired during the loading history. First, damage growth is analyzed by tracking the increase in rms$\left(\varphi_{C}^{P-\mathrm{DVC}}\right)$ with respect to the base level. The rms projection residual levels are divided by that of scan 000 (prior to loading). This is the so-called amplification factor shown in Figure 8.

For $\theta =0^{\circ }$, there is no increase that may reveal damage growth, except for the very last loading step prior to specimen failure, which may also be attributed to the extrapolation using the DVC displacement field of scan 007. The same conclusions are drawn for $\theta =56^{\circ }$. Furthermore, the amplification levels when $\theta =75^{\circ }$ reveal the first significant increase in projection residuals compared to the base level for time step 941, which corresponds to the range of scan 007 (Figure 2b). This observation is consistent with DVC results at a finer scale [34].

The accumulation of damage and the activation and deactivation of cracks (due to the cyclic loading history) is most visible for $\theta =105^{\circ }$. It is concluded that damage within element #89 first initiated at time step 331, which corresponds to the range of scan 003 (Figure 2b). During the subsequent unloading (time step 485), it appears that damage is deactivated. Damage within element #89 is once again active upon loading the specimen to the peak of the third loading cycle (time step 639). However, when the specimen is once again unloaded (time step 793), the crack within element #89 is only partially closed (the amplification of time step 793 is greater than that of time step 485). Within the range of scans 007 and 008 (time steps 947 to 1255) the crack is again active and more pronounced compared to the previous loading stages. The subsequent unloading (time step 1332), once again leads to partial crack closure.

**Figure 9:**
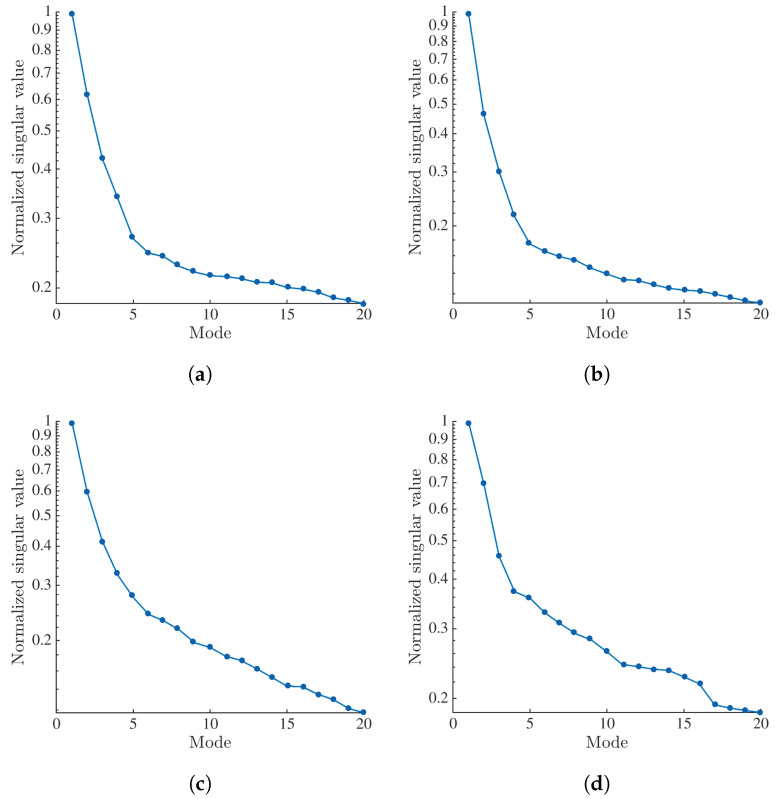
Normalized singular values for element #89 when (**a**) θ=0°, (**b**) θ=56°, (**c**) θ=75° and (**d**) θ=105°.

A singular value decomposition (SVD) is applied to the projection residuals constructing the matrix [U] for each analyzed angle (θ=0°, 56°, 75° and 105°) as explained in Appendix B. The singular values are shown in Figure 9. For all four analyzed angles, the first singular value is significantly higher than the second one, thereby indicating a good separation between them. Thus, only the first temporal mode of each angle is analyzed for the purpose of quantification of damage growth.

**Figure 10:**
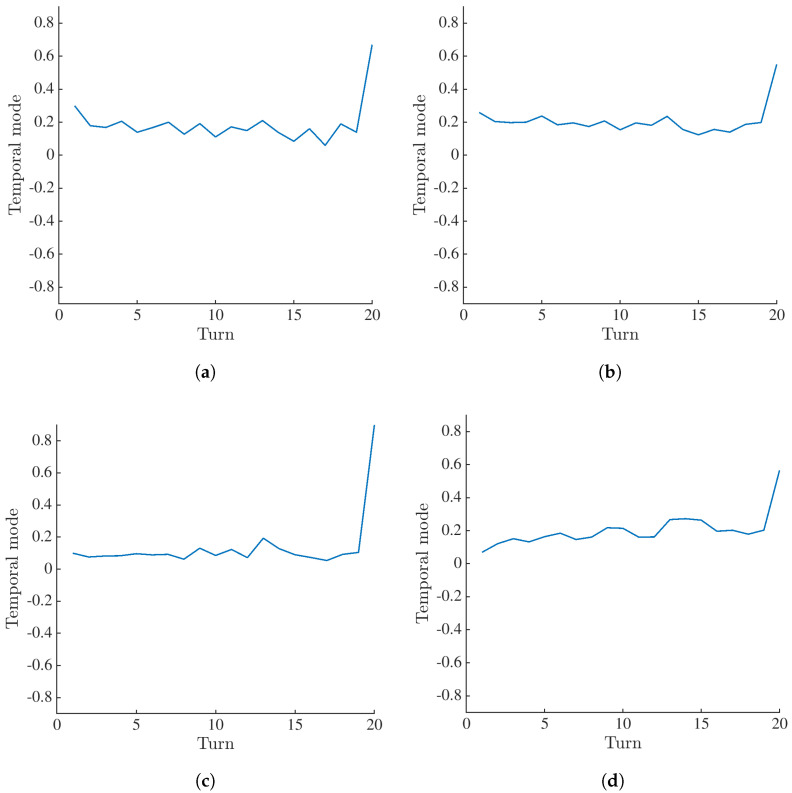
First temporal mode of projection residuals within element #89 computed when (**a**) θ=0°, (**b**) θ=56°, (**c**) θ=75° and (**d**) θ=105°.

Figure 10 shows the first temporal modes calculated when θ=0°, 56°, 75° and 105°. For θ=0° and 56°, the values for different loading steps (in this case marked with turns) are distributed within the same range. Thus, for these angles, damage is not detected. The increases in values for the last turn prior to specimen failure mainly stem from the extrapolation of the DVC field of scan 007. Furthermore, the values obtained for θ=75° reveal the first increase for the loading stage corresponding to scan 005 (turns 9 and 10). However, for subsequent unloading the crack is only partially closed. This remark is consistent with DVC results on a finer scale [34]. Damage growth is pronounced for scans 007 and 008 (turns 13 and 14). The subsequent unloading resulted in partial crack closure. The values reported for θ=105° clearly highlight the accumulation of damage within element #89. These values also reveal the activation and deactivation of damage. Thus, this angle is deemed the most sensitive to the presence of cracks.

**Figure 11:**
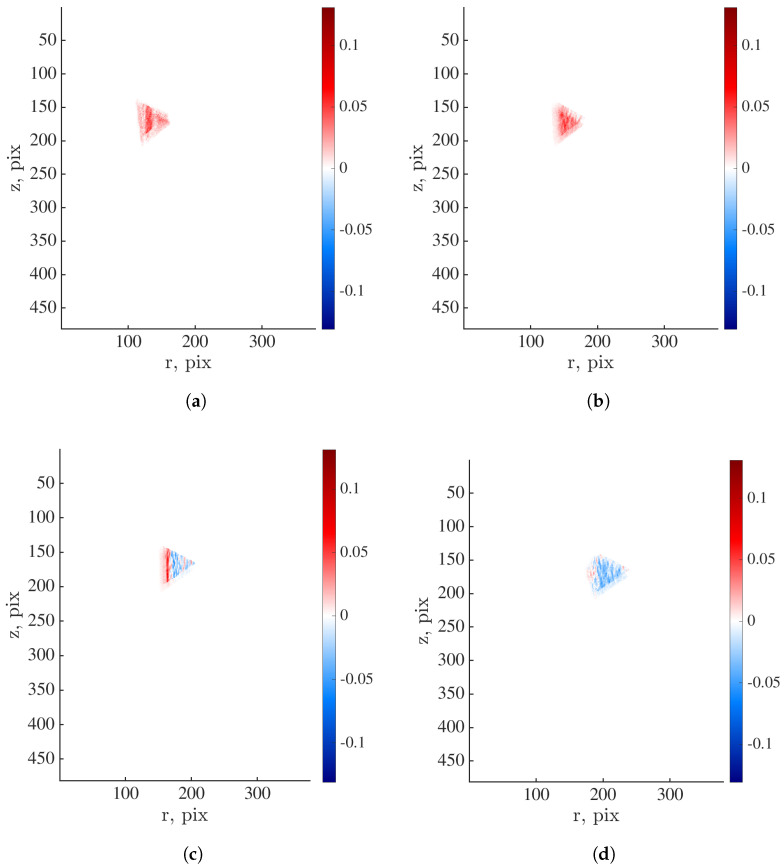
First spatial mode of projection residuals within element #89 calculated when (**a**) θ=0°, (**b**) θ=56°, (**c**) θ=75° and (**d**) θ=105°.

The first spatial modes of the projection residuals computed for the selected angles are shown in Figure 11. For θ=0°, 56° and 105°, the spatial distribution is homogeneous. The area of elevated values is visible near the element angle when θ=75°. The reported results demonstrate that the analysis of projection residuals enables for the quantification of damage growth within the investigated specimen. However, due to the heterogeneous and random mesostructure, the damage analysis is highly dependent on the analyzed angles.

## 5. Conclusions

In the present work, a 4D characterization of a glass fiber mat reinforced polyester resin was performed by employing DVC enhanced P-DVC that relied on spacetime discretization of the measured displacement fields. An alternative approach in which spatial modes were fully constructed with DVC, and only temporal modes were sought via P-DVC was implemented. All the analyses were performed with a temporal basis consisting of 11 plateau-like functions interpolating the cyclic loading history. These temporal shape functions were linked to 11 spatial modes constructed via DVC (i.e., by performing volumetric correlations between scan 0 and all subsequent reconstructed volumes).

A methodology to quantify damage growth was proposed by analyzing projection residuals for specific angles. First, zones in which damage growth was to be quantified were selected based on the DVC gray level residuals. Finite elements exhibiting correlation residuals greater than 70% of the maximum value within the entire ROI composed the mask. This mask was then projected onto the detector plane. In the present study, the analysis of damage growth was performed for one element that contained elevated correlation residuals corresponding to crack propagating from the notch root. The quantification of damage growth was performed by multiplying the projection residuals of this element with the mask of this element. As the temporal sampling was such that 1491 radiographs were analyzed with P-DVC, not all angles were inspected. To find for which angles damage was most pronounced, the DVC gray level residuals of the same element were projected onto the detector plane. Based upon the root mean square residual, specific angles were analyzed. Damage growth was first analyzed by tracking the increase in terms of rms values with respect to the base level. An amplification factor that revealed the increase in residual levels during the cyclic loading history indicated the activation and deactivation of damage. Last, an SVD procedure was also applied to the projection residuals. It was shown that the analysis of projection residuals may enable for the quantification of damage growth within the investigated specimen.

One of the principal aspects of future investigations will be to modify the temporal sampling in a way to find angles most sensitive to cracks. In such a way, the angles that do not display damage would be excluded from the analysis. Further, the presented results were obtained using coarse grained images (i.e., the resulting definition was down-sampled over a 4×4 regular grid). This binning level may lead to a loss of information beneficial for mechanical characterization. Another aspect will thus be to perform 4D measurements at the original scale. In addition, the measurements may be improved by further adjusting the geometrical parameters of the tomography setup, as well as performing beam hardening corrections (which was not carried out in the current work).

The P-DVC procedure employed in the present work offers the opportunity to continuously measure 4D (i.e., 3D space and 1D time) displacement fields with simultaneous loading and rotation of specimens. In the proposed framework, the spatial modes were constructed with scan-wise DVC analyses, and only temporal modes were sought via P-DVC. The P-DVC enhanced DVC method enabled for the quantification of damage growth over the entire loading history up to failure. The continuous acquisition of radiographs over the entire loading history and the analysis of only one radiograph per loading step significantly enriched the temporal sampling compared to scan-wise DVC (i.e., by more than two orders of magnitude in the present case). Due to its rich temporal sampling, the proposed framework may also enable for the assessment of time-dependent phenomena such as, e.g., stress-relaxation and crack propagation. It is important to highlight that the employed P-DVC methodology is generic in a way that it can be applied to different materials with various damage mechanisms and behavior, as well as different in situ experimental protocols (when specimens are simultaneously loaded and imaged). In addition to enabling experimental tests to be performed very quickly, it also allows different in situ experimental protocols (designed for standard full-field measurements) to be optimized. This procedure may significantly reduce the quantity of data required for volumetric full-field measurements, while enriching the temporal sampling compared to standard DVC approaches.

## Figures and Tables

**Table 1:** Converged temporal amplitudes obtained when using 11 plateau-like functions as the temporal basis. The * highlights an extrapolation, namely, the temporal interpolation function describing the last loading stage prior to failure is related to the spatial mode of scan 007.

Scan	Mechanical State	α
000	Unloaded	1.005
001	Loaded	1.007
002	Unloaded	0.983
003	Loaded	1.007
004	Unloaded	1.012
005	Loaded	1.002
006	Unloaded	0.998
007	Loaded	1.011
008	Loaded	1.006
009	Unloaded	0.996
007 *	Loaded	1.128

**Table 2:** Comparison of rms differences expressed in vx in *z*-direction between the nodal displacements measured with FE-DVC and projection-based measurements. The comparison is performed for the converged P-DVC displacement fields for the angles at the beginning of each constant stroke stage.

Scan	Mechanical State	*z*
000	Unloaded	0.005
001	Loaded	0.007
002	Unloaded	0.01
003	Loaded	0.007
004	Unloaded	0.012
005	Loaded	0.002
006	Unloaded	0.011
007	Loaded	0.012
008	Loaded	0.011
009	Unloaded	0.011

**Figure A1:**
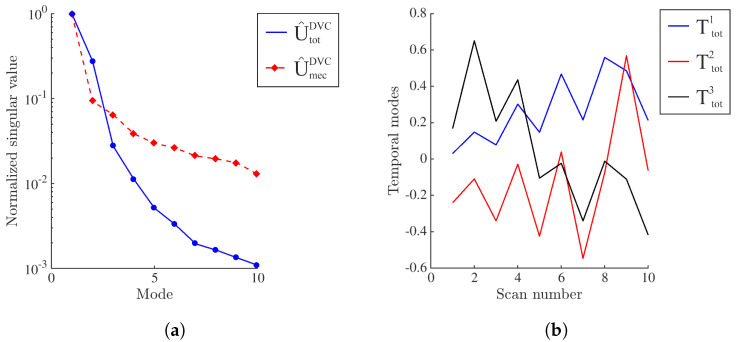
(**a**) Spectrum of normalized singular values. (**b**) First three temporal modes.

**Figure A2:**
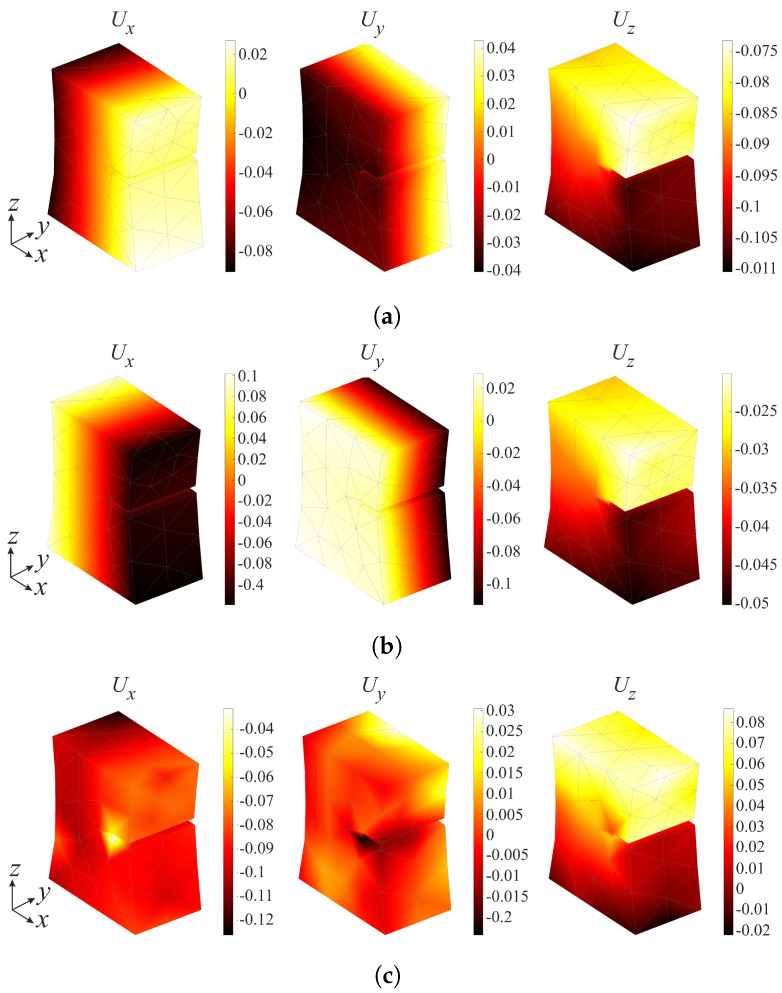
First (**a**), second (**b**), and third (**c**) spatial modes of ${\hat{U}}_{\mathrm{tot}}^{\mathrm{DVC}}$.

**Figure A3:**
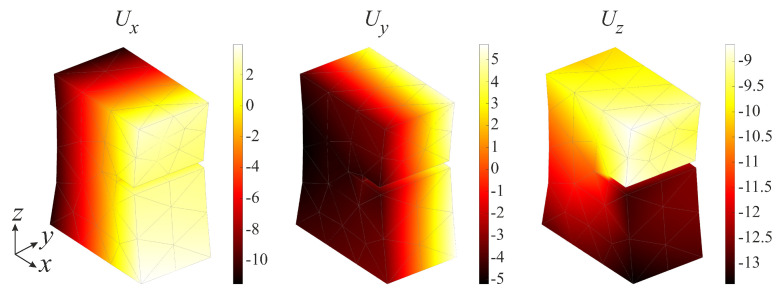
Displacement field of scan 007 containing RBMs.

**Figure A4:**
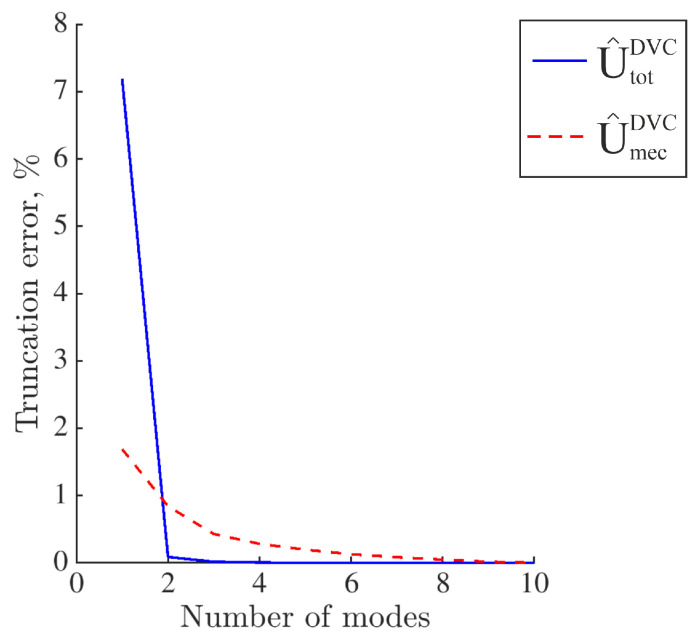
Truncation error as a function of mode number.

**Figure A5:**
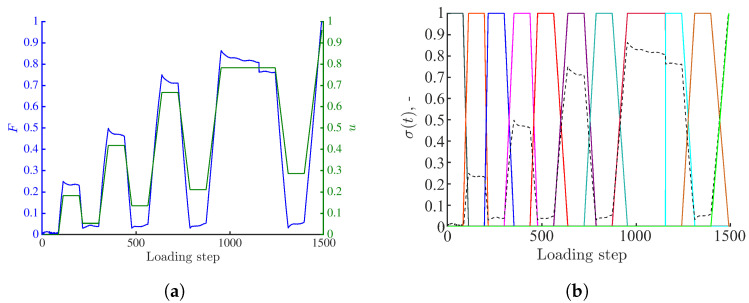
(**a**) Temporal bases consisting of either normalized force history or stroke signal. (**b**) Temporal modes consisting of 11 plateau-like functions. The black dashed line represents the measured force signal normalized by its maximum value.

**Figure A6:**
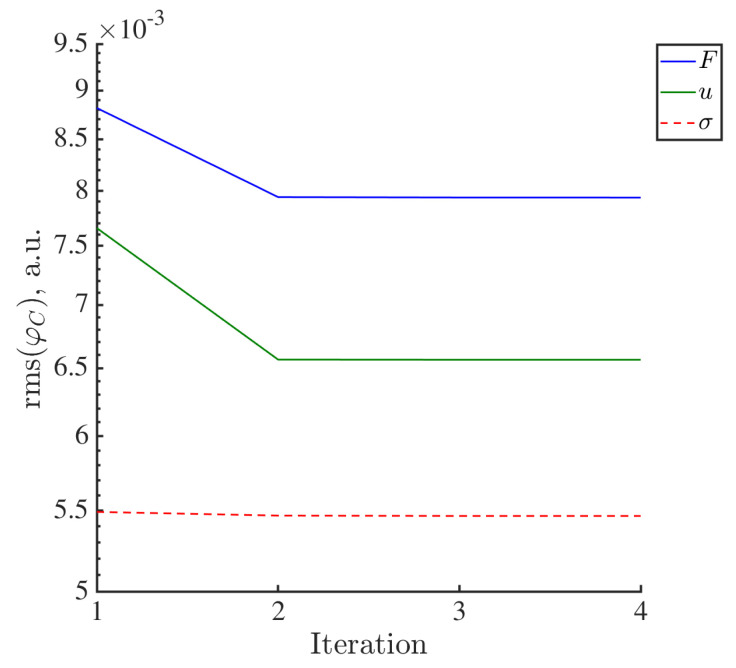
Change in rms projection residuals over the entire Region of Interest during the iterative procedure for different temporal interpolations.

**Figure A7:**
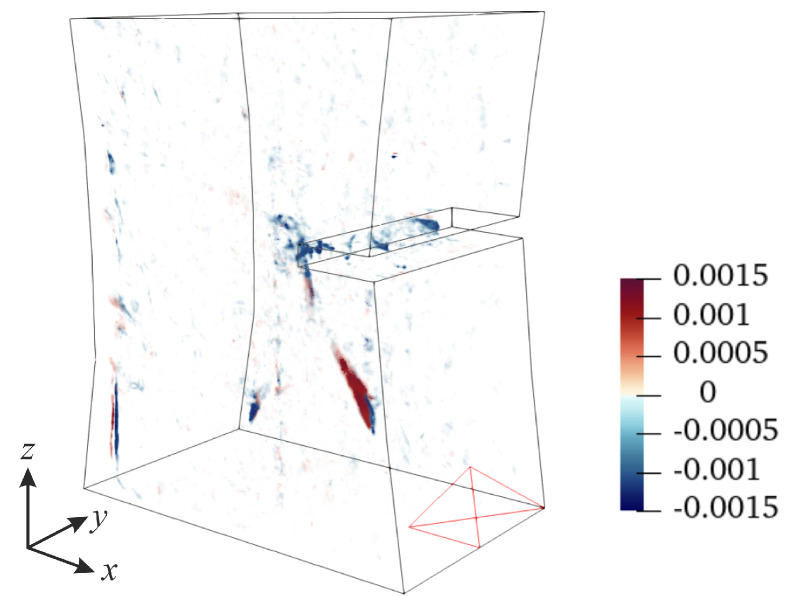
DVC residuals of scan 007. The red contour denotes element #3.

**Figure A8:**
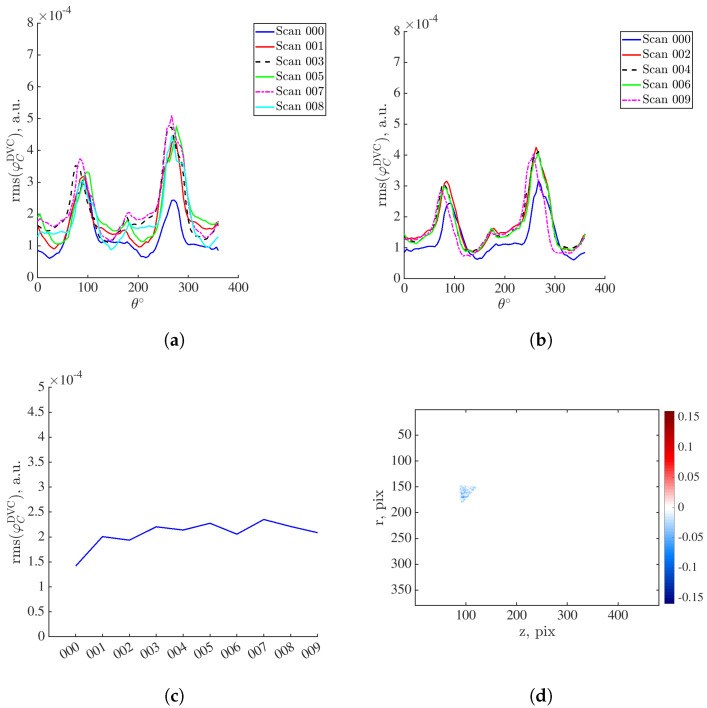
(**a**) Rms DVC residuals for element #3 projected onto the detector plane computed for the scans acquired at the peak of the loading cycles (Figure 2b). (**b**) Corresponding residuals computed for the scans acquired at the unloaded steps (Figure 2b). (**c**) Rms DVC residuals of element #3 during the entire turns of scan acquisitions. (**d**) Projected DVC residuals of element #3 when $\theta =90^{\circ }$.

**Figure A9:**
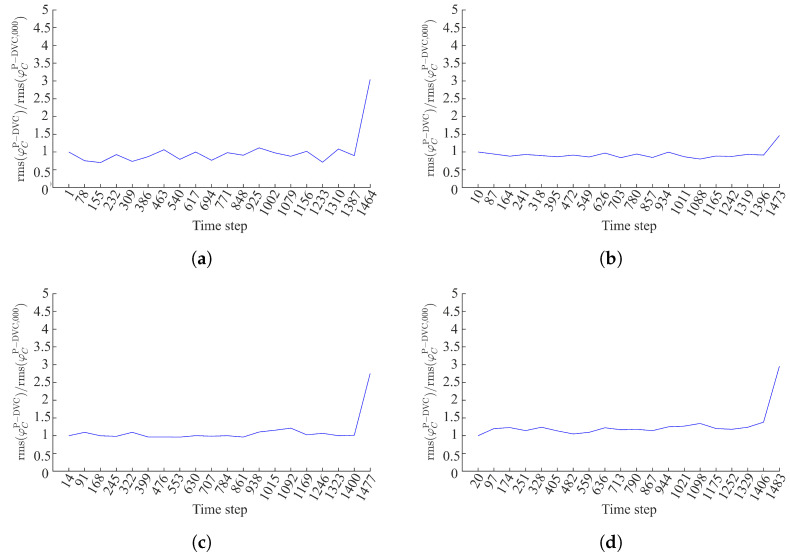
Amplification factor of projection residuals within element #3 when (**a**) θ=0°, (**b**) θ=42°, (**c**) θ=60° and (**d**) θ=90°.

**Figure A10:**
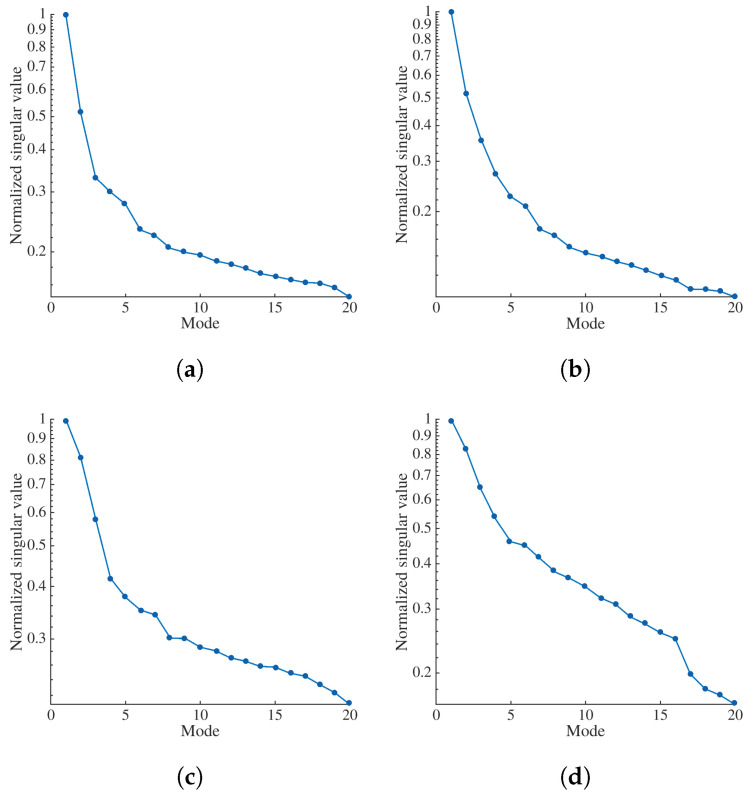
Normalized singular values for element #3 when (**a**) θ=0°, (**b**) θ=42°, (**c**) θ=60°, and (**d**) θ=90°.

**Figure A11:**
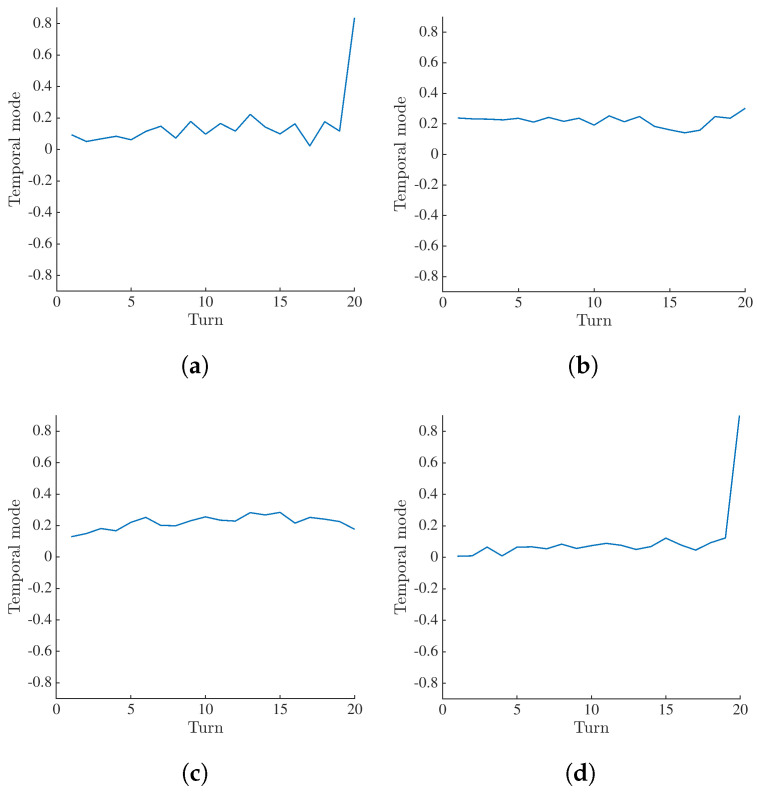
The first temporal mode of projection residuals within element #3 computed when (**a**) θ=0°, (**b**) θ=42°, (**c**) θ=60°, and (**d**) θ=90°.

**Figure A12:**
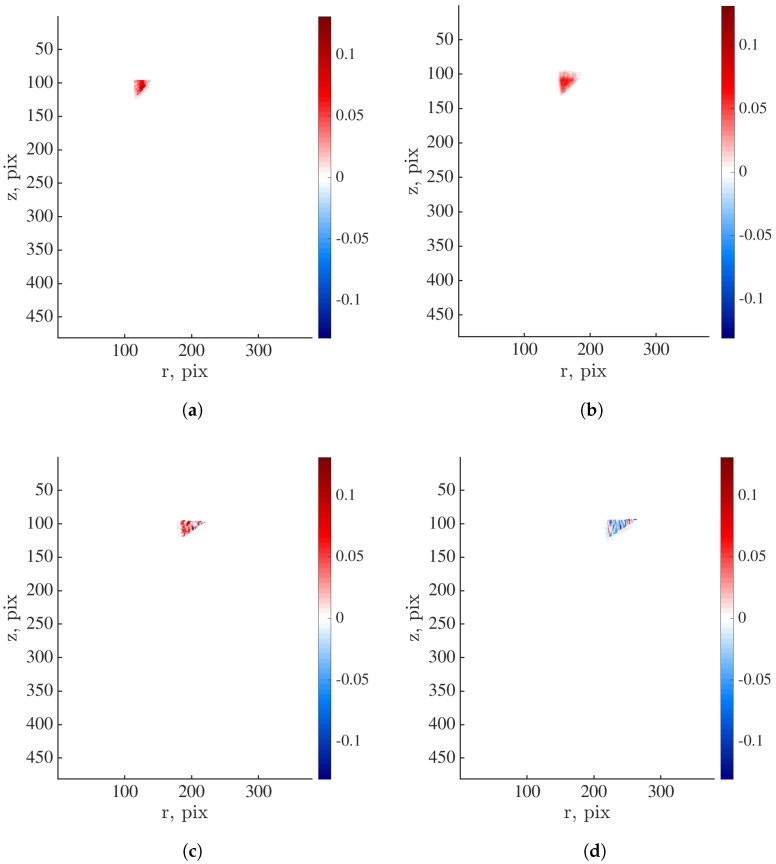
First spatial mode of the projection residuals within element #3 computed when (**a**) θ=0°, (**b**) θ=42°, (**c**) θ=60°, and (**d**) θ=90°.

## Data Availability

The dataset is currently unavailable.

## Abbreviations

The following abbreviations are used in this manuscript:

DVC	Digital Volume Correlation
FE	Finite Element
FDK	Feldkamp–Davis–Kress
FRP	Fiber Reinforced Polymer
HQ	High Quality
P-DVC	Projection-based Digital Volume Correlation
RBM	Rigid Body Motion
RBR	Rigid Body Rotation
rms	Root mean square
ROI	Region Of Interest
SIRT	Simultaneous Iterative Reconstruction
SVD	Singular Value Decomposition
TTC	Tension, Torsion and Compression
XCT	X-ray Computed Tomography
4D	Four-dimensional (3D space and 1D time)

## Appendix A. Hardware Parameters

**Table A1:** Scanning parameters.

Tomograph	North Star Imaging X50+	
X-ray source	XRayWorX XWT-240-CT	
Target/Anode	W (reflection mode)	
Filter	None	
Voltage	150 kV	
Current	7.8 µA	
Focal spot size	5 µm	
Tube to detector	910 mm	
Tube to object	53 mm	
Detector	Dexela 2923	
Definition	1944 × 1536 pixels (2 × 2 binning)	
Scanning settings	High quality	Continuous
Number of projections	800	768
Angular amplitude	360°	360°
Frame average	20 per projection	Continuous (1 per step)
Frame rate	3 fps	3 fps
Acquisition duration	1 h 46 min 26 s	4 min 26 s
Reconstruction algorithm	SIRT	SIRT
Field of view	7.3 × 10 × 20.5 mm^3^	7.3 × 10 × 20.5 mm^3^
Image scale	14.6 µm/voxel	14.6 µm/voxel

## Appendix B. Selection of Temporal Bases

In this appendix, the selection of a suited temporal basis is discussed. This analysis is first conducted by performing a singular value decomposition (SVD) on the DVC displacement fields. An SVD is applied to rectangular matrices ${\left[U\right]}_{m\times n}$, with m>n in this work. To construct this matrix, the nodal displacements of each DVC result are gathered in a column vector, and then concatenated to construct a rectangular matrix [U] that corresponds to a space/turn separation of the measured data.

The mesh consists of 88 nodes (with 3 DOFs per node), and 10 DVC fields are used (namely, scans 000 to 009). Therefore, a 264×10 [U] matrix is created. Three matrices are generated after conducting SVD: the singular values matrix ${\left[S\right]}_{m\times n}$, and the modal matrices ${\left[V\right]}_{m\times m}$ and ${\left[T\right]}_{n\times n}$. These matrices allow for the reconstruction of matrix [U]

(A1) $$\left[U\right]=\left[V\right]\left[S\right]{\left[T\right]}^{⊤},$$

where [S] is a rectangular matrix, and the non-zero terms of this matrix are the singular values $s_{i}$ of the matrix ordered by importance ($s_{1}\geq s_{2}\geq s_{3}\geq \ldots s_{n}$). Due to their ranking, this matrix indicates that certain modes are more significant than others by several orders of magnitude. The spatial modes (SM) are gathered in matrix [V], while the temporal modes (TM) are gathered in [T]. These modes can be truncated to obtain approximations $\left[U_{k}\right]$ with the first *k* instead of all *n* modes

(A2) $$\left[U_{k}\right]=\sum_{j=1}^{k}s_{j}\left\{V_{j}\right\}{\left\{T_{j}\right\}}^{⊤},$$

where $s_{j}$ is the *j*-th singular value $\left\{V_{j}\right\}$ and $\left\{T_{j}\right\}$ is the corresponding modal vectors.

Furthermore, the displacement fields measured with DVC consisted of rigid body motions (RBMs) $U_{\mathrm{rbm}}^{\mathrm{DVC}}\left(\hat{x},T\right)$ and the part causing the deformation called mechanical displacement $U_{\mathrm{mec}}^{\mathrm{DVC}}\left(\hat{x},T\right)$

(A3) $${\hat{U}}_{\mathrm{tot}}^{\mathrm{DVC}}\left(\hat{x},T\right)={\hat{U}}_{\mathrm{rbm}}^{\mathrm{DVC}}\left(\hat{x},T\right)+{\hat{U}}_{\mathrm{mec}}^{\mathrm{DVC}}\left(\hat{x},T\right).$$

The SVD procedure is applied to both ${\hat{U}}_{\mathrm{tot}}^{\mathrm{DVC}}$ (i.e., the displacement fields containing RBMs) and ${\hat{U}}_{\mathrm{mec}}^{\mathrm{DVC}}$ (i.e., the displacement fields from which RBMs are subtracted). This analysis is performed by using the displacement fields of scans 000 to 009. Figure A1a shows the spectrum of singular values normalized by the highest one (i.e., $s_{1}$). From the reported data, it is shown that for both analyzed cases $s_{1}$ is greater than $s_{2}$ by about one order of magnitude, which indicates that Mode 1 captures most of the 4D kinematics. In the case of ${\hat{U}}_{\mathrm{tot}}^{\mathrm{DVC}}$, the difference between the first two singular values was smaller compared to ${\hat{U}}_{\mathrm{mec}}^{\mathrm{DVC}}$. This may be attributed to the influence of RBMs. It is worth highlighting that large RBMs are observed during the experiment, and hence they participate actively to the first mode of ${\hat{U}}_{\mathrm{tot}}^{\mathrm{DVC}}$.

The temporal modes obtained with ${\hat{U}}_{\mathrm{tot}}^{\mathrm{DVC}}$ are shown in Figure A1b. From $T_{\mathrm{tot}}^{1}$, a clear temporal signature resembling the cyclic loading history is observed (Figure 2b). Because of the orthogonality requirement of SVD, the fluctuations for higher order modes are more difficult to interpret.

The first three spatial modes of ${\hat{U}}_{\mathrm{tot}}^{\mathrm{DVC}}$ are shown in Figure A2. In the first two spatial modes, large rigid body rotations (RBRs) are observed in the in-plane components, which occurred during the experiment. For the loading direction, the displacements of the first two modes correspond to tensile loading of the notched sample. The third mode is much more difficult to elucidate.

The first two modes are compared to the displacement field of scan 007 (Figure 2b), as shown in Figure A3. The first mode is easily interpreted as the dominant kinematic mode.

To confirm the previous statement, Table A2 gathers rms differences between the DVC displacement field of scan 007 and rescaled 1st, 2nd, and 3rd spatial modes. The difference for the first spatial modes is very small compared to those of the two higher modes.

**Table A2:** Rms difference expressed in vx between the DVC displacement field of scan 007 and rescaled 1st, 2nd, and 3rd spatial mode.

	*x*	*y*	*z*
1st mode	0.34	0.08	0.09
2nd mode	4.6	2.0	1.2
3rd mode	4.4	2.7	7.8

Last, the truncation error evaluates the loss of information caused by using a subgroup of modes to reconstruct the kinematics. It is computed as

(A4) $$\mathrm{Truncation}\;\mathrm{error}=\frac{∥\left[U\right]-\left[U_{k}\right]{∥}^{2}}{{∥\left[U\right]∥}^{2}}.$$

Figure A4 shows the truncation error as a function of mode number. If only one mode is kept, the truncation error is significantly larger compared to the error if more modes are kept. For the first mode, when the total displacement field is considered, the truncation error is significantly higher than that of the mechanical field. This difference is due to significant RBRs. Since the truncation error when only 1 mode is kept is not too high for the total displacement fields, it is decided to investigate two reduced bases consisting of one single mode. Both of them are compared to results obtained with the full set of DVC displacements.

Different temporal interpolations are exploited on the chosen temporal sampling. First, the 4D kinematics is measured using the normalized force signal as a unique temporal mode (Figure A5a). The measured uniaxial force history reveals rapid but limited relaxations during the acquisition of scans 001, 003, and 005 (the drop of the peak force is approximately 160 N, see Figure 2b). During the acquisition of scans 007 and 008, the force decrease is approximately 50 N and 20 N, respectively.

Next, the normalized stroke is used as a unique temporal mode (Figure A5a). In both cases, only one DVC field is used as spatial mode, namely, the total displacement field of scan 007 (corresponding to the last loading cycle prior to specimen failure). This field is obtained by running DVC between HQ scan 0 (reference) and scan 007. It is important to emphasize that 4D measurements are performed until specimen failure (i.e., after acquiring scan 007). This extrapolation is justified by the fact that scan 007 is acquired at the peak of the last loading cycle prior to specimen failure.

The third approach consists in using 11 plateau-like functions interpolating the cyclic loading history (Figure A5). These temporal shape functions are linked to 11 spatial modes. The latter ones are constructed via DVC, i.e., by performing volumetric correlations between scans 0 (reference scan) and all subsequent reconstructed volumes (Figure 2b). It is important to emphasize that all P-DVC measurements are performed until specimen failure (i.e., after acquiring scan 009). The last temporal mode is related to the DVC field of scan 007. The (small) force drop between scans 007 and 008 is due to the fact that the continuous acquisition between these two steps was interrupted by acquiring one high-quality scan, which is excluded from the present analyses.

The initial value of the amplitude vector $\left\{\alpha^{0}\right\}$ is set to 1 (Equation (7)). In all three cases, the P-DVC analysis takes four iterations to converge. Each iteration lasts approximately 5 h. The measured displacements yield a global decrease of the residuals within the investigated ROI, as shown in Figure A6. The rms projection residuals within the ROI are the lowest when using 11 plateau-like functions. From the reported results, it is shown that utilizing only one DVC displacement field (as a spatial mode) is sufficient to properly capture the kinematics up to specimen failure.

Furthermore, the rms differences between the 3D nodal displacements obtained with FE-based DVC and projection-based measurements are calculated. Since large RBRs in *x*- and *y*-directions are observed during the experiment, these differences are only calculated for the *z*-direction displacements as the mechanical component is dominant in that direction. The rms differences are calculated between DVC displacement fields and converged P-DVC displacement fields for the time-steps corresponding to the beginning of each constant stroke stage. The obtained values are gathered in Table A3. As the projections acquired at the maximum load levels are affected by time-dependent motions (i.e., stress-relaxation phenomena), which influence the reconstruction of the full volume, differences are expected. From the values reported in Table A3, it is concluded that, when using measured force or prescribed stroke history as a temporal basis in combination with a single spatial mode, the rms differences are higher for the unloaded stages. This is especially pronounced when using the measured uniaxial force as the temporal interpolation basis, due to the fact that part of the deformation mechanisms is deactivated [34]. For the loaded stages, the differences are very small. When employing 11 plateau-like temporal functions, the rms differences are very small. The reported values confirm that, despite using only one spatial mode (i.e., the displacement field of scan 007) when performing 4D measurements, the kinematics is very well captured. In addition, employing the stroke history as the temporal interpolation better captured the temporal fluctuations (as the rms differences are smaller compared to those obtained when using the measured uniaxial force). However, the rms projection residuals within the ROI are the lowest when using 11 plateau-like functions. With such settings, the kinematics of the specimen is properly captured. This observation is also consistent with the truncation analysis where it was shown that the truncation error is significantly lower if more modes are kept (compared to the error if only one mode is used, see Figure A4).

**Table A3:** Comparison of rms differences in *z* direction between the 3D nodal displacements measured with FE-DVC and projection-based measurements. The comparison is performed for the converged P-DVC displacement fields for the angles at the beginning of each constant stroke stage.

		*F*	*u*	σ
**Scan**	**Mechanical State**	z	z	z
000	Unloaded	0.05	0.05	0.005
001	Loaded	0.81	0.05	0.007
002	Unloaded	0.35	0.06	0.01
003	Loaded	0.88	0.06	0.007
004	Unloaded	1.56	0.22	0.012
005	Loaded	0.73	0.08	0.002
006	Unloaded	2.77	0.3	0.011
007	Loaded	0.78	0.09	0.012
008	Loaded	0.76	0.08	0.011
009	Unloaded	3.76	0.25	0.011

Table A4 gathers the converged amplitudes. The amplitudes obtained with normalized force or stroke signals are very close. In both cases, the temporal amplitudes are greater than 1, as the DVC displacement field of the last loading cycle prior to specimen failure is used as spatial mode, while the 4D characterization is performed beyond this stage (i.e., up to specimen failure). Despite this extrapolation, the displacement fields for all analyzed radiographs converge and are consistent. When using 11 plateau-like functions, the amplitudes are close to 1 for all loading steps, except for the last one where the amplitude is greater than 1. Once again, this result stems from the fact that the DVC displacement field of scan 007 (Figure 2) is used as spatial component of the last mode, whereas 4D kinematic measurements are performed up to specimen failure (beyond scan 007). The displacement fields measured with P-DVC beyond scan 009 converge and are consistent. From the reported data, it is concluded that the kinematics is very well captured with the chosen temporal sampling, even though the displacement field becomes more complex with the increase in load level. Based on the reported data, 11 plateau-like functions constrained to 11 spatial modes obtained with FE-DVC are chosen as a suited temporal basis (Section 3).

**Table A4:** Temporal amplitudes. The * highlights an extrapolation, namely, when employing 11-plateau like functions as temporal basis, the temporal interpolation function describing the last loading stage prior to failure was constrained to the spatial mode of scan 007. When using the measured uniaxial force or stroke signals, only the displacement field of scan 007 is considered as the spatial mode.

		*F*	*u*	σ
**Scan**	**Mechanical State**	α	α	α
000	Unloaded			1.005
001	Loaded			1.007
002	Unloaded			0.983
003	Loaded			1.007
004	Unloaded			1.012
005	Loaded			1.002
006	Unloaded			0.998
007	Loaded			1.011
008	Loaded			1.006
009	Unloaded			0.996
007 *	Loaded	1.206	1.197	1.128

## Appendix C. Validation of the Methodology for Quantification of Damage Growth

To validate the damage detection procedure (Section 4), an element within which damage does not occur is analyzed in the sequel. It corresponds to element #3, which is located at the specimen bottom surface (Figure A7).

The DVC residuals within element #3 are projected onto the detector plane (Figure A8a,b). The first interval of residual increase for the loaded scans is within the range θ=50° to 110°. The reported values are lower than those obtained within element #89 (Figure 7). There is an increase in correlation residuals with respect to levels of scan 000. However, it is not as pronounced as for element #89. It is also important to note that the residual levels obtained for the scans acquired in the loaded steps lie within the same range (Figure A8a). The values obtained for unloaded scans are within the range of scan 000 (Figure A8b). When plotting the rms DVC residuals of element #3 during the entire turns of scan acquisitions, the correlation residuals remain within the same range (Figure A8c). The projected residual map within element #3 calculated for scan 007 when $\theta =90^{\circ }$ is shown in Figure A8d. Their distribution is homogeneous, and the levels are low. It is concluded that damage did not occur within this element. This hypothesis will also be inspected with the projection residuals.

As the first interval of DVC residual increase is within the range θ=50° to 110° (Figure A8), the projection residuals are thus only inspected for specific angles, namely, θ=0, 42°, 60° and 90° acquired during the loading history. Figure A9 shows the amplification factor for these angles. For all observed angles, the values for the whole loading history are within the same range. There are no significant increases in amplification levels that may highlight damage growth, except for the very last part of the experiment.

The SVD procedure is also applied to the projection residuals for each analyzed angle (θ=0°, 42°, 60°, and 90°) as explained in Section 4. The singular values are shown in Figure A10. For all four analyzed angles, the first singular value is higher than the second one, thereby indicating a good separation between them. Thus, only the first temporal mode of each angle is analyzed.

Figure A11 shows the first temporal modes computed for θ=0°, 42°, 60°, and 90°. When θ=42° and 90°, the levels are within the range of the unloaded stage (here marked with turn 1, corresponding to the range of scan 000). However, increases in levels compared to the first turn are observed when θ=0° and 60°. This trend is due to the fact that these angles may be impacted by damage occurring in other zones/elements. Still, these increases are deemed not significant. The reported results demonstrate that, in this specific case, the damage analysis is highly dependent on the analyzed angles.

Last, the first spatial modes of the projection residuals computed for the chosen angles are shown in Figure A12. For all analyzed angles, the spatial distribution within element #3 is homogeneous.
